# Comparative outcomes of conventional follitropin delta 15 µg/day versus follitropin alfa 225 IU/day for ovarian stimulation: results from an exploratory *post hoc* analysis of Spanish participants from the ADAPT-1 randomized controlled trial

**DOI:** 10.3389/frph.2026.1927550

**Published:** 2026-09-30

**Authors:** Marta García Sánchez, Andrea Bernabeu, Ana Hernández Cesteros, Laura Blasco Gastón, Sofia Ortega Ricondo, Gemma Castillón, Marcos Ferrando Serrano, Signe Møgelmose, Enrica Gravotta, Rita Lobo

**Affiliations:** 1 IVIRMA Global Research Alliance, IVIRMA, Málaga, Spain; 2 Hospital Quironsalud Málaga, Málaga, Spain; 3 Instituto Bernabeu Alicante, Albufereta, Alicante, Spain; 4 Vida Recoletas Salud, Valladolid, Spain; 5 Ginefiv, Madrid, Spain; 6 Hospital Universitario Principe De Asturias, Madrid, Spain; 7 IVI, Barcelona, Spain; 8 IVIRMA Global Research Alliance, IVIRMA, Bilbao, Spain; 9 Global Biometrics, Ferring Pharmaceuticals, Kastrup, Denmark; 10 Global Research and Medical, Ferring Pharmaceuticals, Kastrup, Denmark

**Keywords:** conventional dosing regimen, FE999049, follitropin delta, gonadotropins, normo-responders, ovarian stimulation, rFSH

## Abstract

**Research question:**

Can follitropin delta be used in a conventional regimen (15 µg/day starting dose) without compromising efficacy and safety in patients seeking fertility treatment in Spain?

**Design:**

This was an exploratory *post hoc* analysis of the ADAPT-1 randomized controlled trial. Participants, 18–40 years, enrolled at specialist reproductive centers in Spain in this accessor-blinded, multicenter trial assessing efficacy and safety of conventional follitropin delta 15 μg/day and follitropin alfa 225 IU/day for ovarian stimulation. The primary endpoint was the number of oocytes retrieved. During the follow-up period, clinical pregnancies from the first fresh or frozen transfer within 3 months of the start of stimulation, and ovarian hyperstimulation syndrome (OHSS) rates were assessed. All cycles used a gonadotropin-releasing hormone (GnRH) antagonist protocol.

**Results:**

A total of 132 patients were randomized (10-AUG-2022 to 11-MAR-2024): follitropin delta group, *n* = 85; follitropin alfa group, *n* = 47. The adjusted mean number of oocytes retrieved was 10.6 and 11.0 for each group, respectively [difference: −0.3 (95% CI: −2.4; 1.7)]. Adjusted clinical pregnancy rates per started cycle were 34.8% and 32.0%, respectively [difference: 2.8% (95% CI: −14.3; 20.0)]. Adverse events were reported for 28.2% and 42.6% of participants, respectively, with the most common adverse events being headache [10.6% (*n* = 9) and 8.5% (*n* = 4)], pelvic discomfort [4.7% (*n* = 4) and 4.3% (*n* = 2)], dysmenorrhea [0% and 6.4% (*n* = 3)], and early OHSS [1.2% (*n* = 1) and 4.3% (*n* = 2)].

**Conclusions:**

Our exploratory analysis shows that a higher starting dose of follitropin delta 15 µg/day in a conventional regimen can be successfully used in appropriately selected infertile patients, resulting in a good average oocyte yield without compromising first-transfer clinical pregnancy rates and while maintaining a low incidence of early OHSS.

## 1 Introduction

Spain is a major provider of assisted reproductive technologies (ART) in Europe and a common destination for cross-border reproductive care (1, 2). In Spanish clinical practice, recombinant follicle-stimulating hormone (rFSH) dosing is normally individualized according to ovarian reserve and predicted ovarian response (3, 4). As such, there is a strong clinical relevance for evaluating ovarian stimulation and reproductive outcomes among participants enrolled at Spanish sites in the multinational ADAPT-1 trial (5).

Ovarian stimulation is an essential part of IVF/ICSI, with rFSH most commonly recommended in Spain as part of a gonadotrophin-releasing hormone (GnRH) antagonist protocol for normal or hyper-responding patients under 35 years old (6). In line with current guidelines, clinicians in Spain offer individualized dosing strategies for rFSH ranging between 150 IU and 225 IU for predicted normal responders, taking account of the patient's ovarian reserve and predicted or previous ovarian response, with the goal of simultaneously maintaining the patient's safety while obtaining the optimal number of oocytes (3, 4).

Follitropin alfa and follitropin delta are rFSH preparations which both have an identical amino acid sequence to endogenous human FSH, but post-translational glycosylation patterns of follitropin delta are more similar to endogenous human FSH compared with follitropin alfa (7). The registration trials for follitropin delta used a fixed daily dose that had been algorithmically calculated based on serum anti-Mullerian hormone (AMH) and body weight as part of a GnRH antagonist protocol (8–10). In contrast, other approved rFSH preparations, including follitropin alfa, are conventionally dosed at 150–225 IU for the first 4–6 days in women who are predicted normal responders, with potential subsequent dose adjustments (maximum daily dose 450 IU).

Previously, the ADAPT-1 trial compared conventionally dosed follitropin delta 15 µg with follitropin alfa 225 IU, fixed for the first 5–6 days, after which dose adjustments could be made, in a GnRH antagonist protocol and showed similar ovarian responses and clinical pregnancy rates between treatments groups (5). ADAPT-1 was a multinational study, recruiting patients from Austria, France, Italy, Spain, and the United Kingdom, with Spanish sites recruiting 132 of the 300 participants. The aim of our study is to report the ovarian responses, clinical pregnancy rates and safety when using conventionally dosed follitropin delta starting at 15 µg/day and follitropin alfa 225 IU/day for women who were suitable for a gonadotropin starting dose of 225 IU/day and seeking medical support to become pregnant at specialist reproductive medicine centers in Spain as an exploratory *post hoc* subgroup analysis of the ADAPT-1 trial.

## 2 Materials and methods

### 2.1 Trial design

This randomized controlled trial was conducted according to the International Council for Harmonisation guideline on Good Clinical Practice (GCP). The trial protocol and all relevant documents were approved by the Institution Ethics Committees for each trial site. All patients provided informed written consent to participate in the trial. Trial registration was with clinicaltrials.gov (NCT05263388) and Eudract (2021-001785-38).

### 2.2 Trial participants

Participant selection followed the eligibility criteria of the parent trial, ADAPT-1 with the additional criterion of enrolment at a Spanish trial center. Infertile women or women with partners diagnosed with male factor infertility, 18–40 years old, who were presumed ovulatory (regular menstrual cycles of 21–35 days) could be enrolled at seven specialist reproduction medicine centers in Spain. Infertility diagnoses were tubal infertility, unexplained infertility, endometriosis stage I/II or partners diagnosed with male factor infertility. Participants ≤37 years had been infertile for at least 1 year whereas participants >37 years had been infertile for at least 6 months prior to enrolment.

All participants were eligible for IVF and/or ICSI using fresh or frozen ejaculated sperm from male partner or sperm donor. Patients with previous history of ovarian hyperstimulation syndrome (OHSS), exuberant ovarian response to gonadotropins or polycystic ovarian syndrome [PCOS, which has recently been re-named as polyendocrine metabolic ovarian syndrome (PMOS)], or were considered unsuitable for a starting dose of gonadotropin 225 IU/day, as judged by the investigator, were excluded from enrolment. The full list of inclusion and exclusion criteria is listed Supplementary Table S1.

### 2.3 Trial endpoints

The primary endpoint was the number of oocytes retrieved. Secondary endpoints comprised total gonadotropin dose and number of stimulation days; follicular development (total and by size category) at end of stimulation; serum estradiol and progesterone concentrations at end of stimulation; number of fertilized oocytes, blastocysts, and good quality blastocysts; implantation rates, and clinical pregnancy rates from the first transfer within 3 months of the start of stimulation assessed per started cycle (pre-specified) and per participant with a blastocyst transfer (post-hoc analysis). Safety and adverse event monitoring was conducted throughout the trial, including early/late OHSS and preventive interventions for early OHSS. We also recorded gonadotropin dose adjustments, number of metaphase II (MII) oocytes and whether the transfer was with a fresh or frozen blastocyst.

### 2.4 Randomization and treatment

Participants were randomized within 90 days of screening to receive either follitropin delta or follitropin alfa in a predefined 2:1 ratio. Consequently, unequal treatment group sizes reflect the planned trial design of ADAPT-1, which is the parent trial of our *post-hoc* subgroup analysis. Participants started stimulation on Day 2 or 3 of their menstrual cycle with a daily dose follitropin delta 15 µg (REKOVELLE®, Ferring Pharmaceuticals, Kastrup, Denmark) or follitropin alfa 225 IU (GONAL-F®, Merck Europe B.V, Amsterdam, The Netherlands). Each gonadotropin was administered using each drug's commercially available pre-filled injection pen. The gonadotropin starting dose was fixed for the first 4 days. Dose adjustments could be made on the day of starting the GnRH antagonist (stimulation Day 5 or 6), or later, and no more frequently than every 2 days. At each dose adjustment, the daily dose could be increased or decreased by 5 µg for follitropin delta or by 75 IU for follitropin alfa. The maximum permitted daily dose was 20 µg for follitropin delta and 300 IU for follitropin alfa.

### 2.5 Trial procedures

Premature luteinizing hormone surge was prevented by daily subcutaneous administration of a GnRH antagonist (ganirelix acetate 0.25 mg; FYREMADEL®, SUN Pharma, Mumbai, India) from stimulation Day 5 or 6 until the end of stimulation. Triggering of final follicular maturation was either by a single subcutaneous injection of human chorionic gonadotropin 250 μg (hCG; OVITRELLE®, Merck, Darmstadt, Germany) if ≥3 follicles with a diameter ≥17 mm (or if the investigator judged that ≥3 follicles with a diameter ≥17 mm could not be reached and that triggering was preferred instead of cycle cancellation) or two consecutive subcutaneous injections of GnRH agonist [relin acetate 0.1 mg (0.2 mg in total) GONAPEPTYL®, Ferring, Kastrup, Denmark] if there were ≥25 follicles with a diameter ≥12 mm or the serum estradiol was ≥5,000 pg/mL (18,355 pmol/L) (local laboratory) or a freeze-all approach was intended. Dual triggering was not allowed. Cycles could be cancelled for poor (if the investigator judged that the triggering criteria could not be met by Day 20 of stimulation) or excessive (judged by the investigator based on local practices) ovarian responses.

Oocyte retrieval took place 36 ± 2 h after triggering of final follicular maturation and were inseminated by IVF and/or ICSI. Fertilization was assessed on Day 1 after oocyte retrieval. Blastocyst culture and Day 5 blastocyst-transfer were mandatory for a fresh transfer. Participants who underwent final follicular maturation with a GnRH agonist could undergo a transfer in a frozen cycle using blastocysts cryopreserved on Day 5 or Day 6 after oocyte retrieval. Blastocyst quality was assessed on the last day of culture using the Gardner and Schoolcraft scoring system (11). Day 5 and Day 6 blastocyst reproductive outcomes were summarized together because the study was not designed or powered to compare outcomes according to the blastocyst development day. Blastocysts that were not transferred in the fresh cycle were cryopreserved in accordance with local guidelines and/or regulations. Reproductive outcomes from fresh and frozen transfers were analyzed together. Luteal phase support in fresh and frozen cycles as well as potential other medicinal products for programming of frozen cycles was in accordance with the site's clinical practice.

#### 2.5.1 Follow-up period transfer procedures

The number of transferred blastocysts in the first fresh or frozen cycle was based on the participant's wishes, the investigator's recommendation and in accordance with local guidelines and/or regulations. Only the first frozen transfer within 3 months of the start of stimulation for each participant was included in the trial.

### 2.6 Laboratory analysis

Serum AMH, estradiol and progesterone were measured using a central laboratory on Day 1 of stimulation, as well as end-of-stimulation for serum estradiol and progesterone.

### 2.7 Statistical analysis

This exploratory *post hoc* subgroup analysis of participants enrolled at Spanish sites was not independently powered, and no formal hypothesis testing was conducted for this subgroup. The results should therefore be interpreted as exploratory and descriptive.

For the primary endpoint (number of oocytes retrieved) and secondary ovarian response endpoints, the mean treatment differences and the associated 95% confidence intervals (CIs) were derived from a negative binomial regression model with treatment and serum AMH level at stimulation Day 1 as factors (AMH <15 pmol/L, AMH ≥15 pmol/L, or missing) using the delta method. The fertilization rate was calculated using the number of retrieved oocytes as the denominator as per the predefined analysis plan; therefore, the reported rate is not directly comparable with fertilization rates calculated only among mature inseminated or injected oocytes. The implantation rate was calculated using a negative binomial regression model with treatment and age group as factors and transferred blastocysts as offset. Clinical pregnancy rates were compared between treatment groups using a logistic regression model with treatment and age group (<35, 35–37 and 38–40 years) as factors. Descriptive (i.e., unadjusted) clinical pregnancy rates are reported by age category and treatment group per started cycle and per participant with at least one blastocyst transfer. Endocrine parameters were analyzed using multiplicative ANCOVA model with treatment and AMH group as factors and baseline serum concentration as covariate. Treatment differences and confidence intervals were derived using the delta method. The number of stimulation days was analyzed using an ANOVA model with treatment and AMH group as factors. Missing observations for oocytes retrieved, fertilized oocytes, number of blastocysts and clinical pregnancy were replaced with “zero” or “not pregnant” (i.e., worst-case imputation technique).

Baseline demographic and clinical characteristics were summarized descriptively. Formal statistical comparisons of baseline characteristics were not performed because participants were randomized and such testing is not recommended in the reporting of randomized clinical trials (12). Descriptive statistics were used to report the distribution of oocytes retrieved, MII oocytes, and OHSS and/or preventive interventions for subgroup strata based on serum AMH level on Day 1 of stimulation (<15 or ≥15 pmol/L).

## 3 Results

### 3.1 Participant enrolment and discontinuations

A total of 147 infertile patients were screened at seven specialist reproductive medicine clinics across Spain between 10 August 2022 and 11 March 2024. Of the 15 participants who were screened but not randomized, 11 did not fulfil the inclusion and exclusion criteria, and 4 had “other” reasons for not enrolling (1 did not give consent, 2 did not return to the clinic within 90 days to be randomized and 1 had an unforeseen family event). Of the screened patients, 132 consented to be enrolled in the trial and were randomized and received their allocated trial drug (follitropin delta group, *n* = 85; follitropin alfa group, *n* = 47; Figure 1). A total of five participants did not complete the study (follitropin delta group: 2 participants had “other” reason for discontinuation; follitropin alfa group: 1 participant withdrew consent, 1 had an adverse event; 1 had “other” reason).

**Figure 1:**
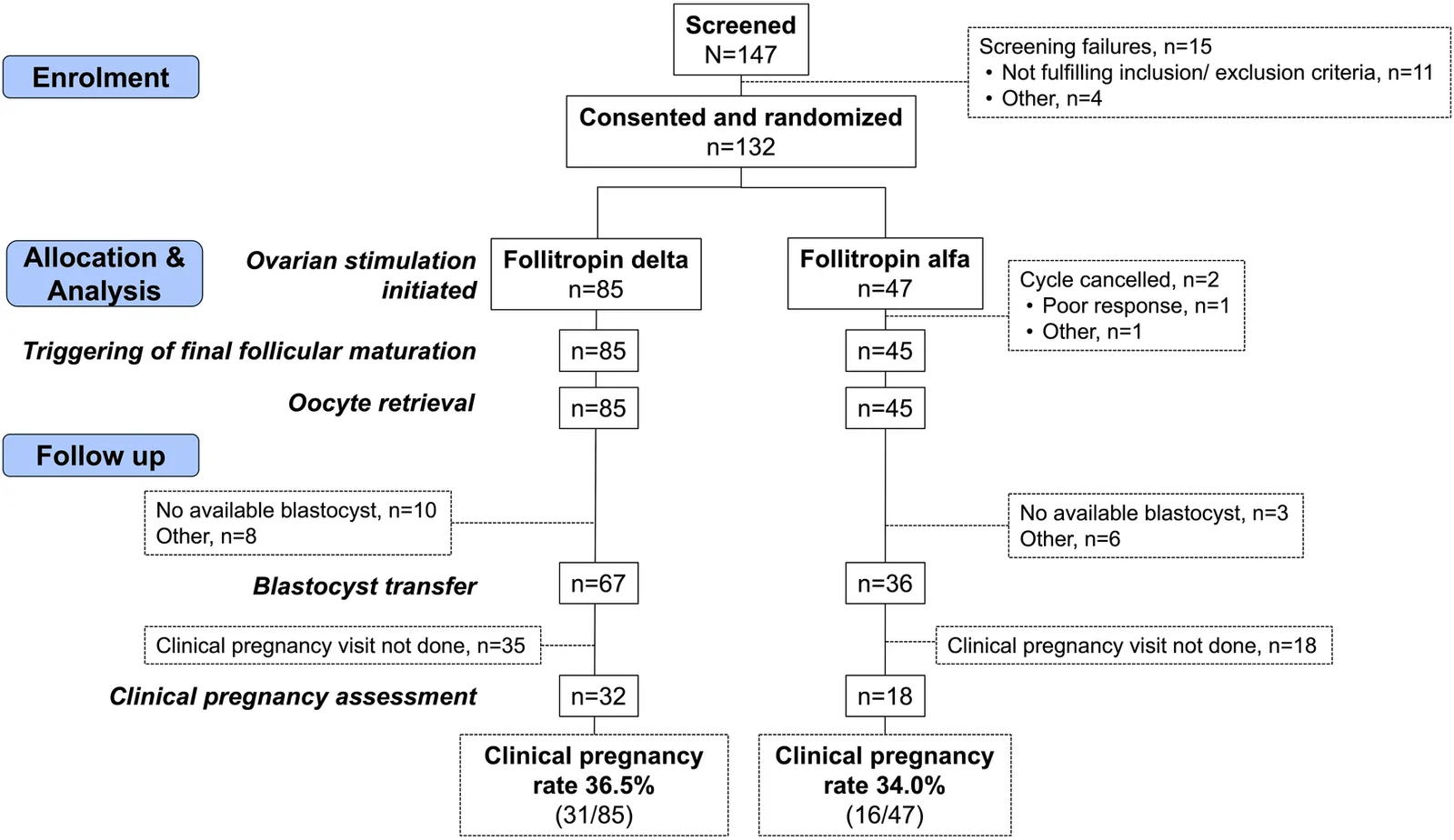
Participant trial flow diagram.

### 3.2 Demographics and baseline characteristics

The demographics and baseline characteristics were mostly similar between the treatment groups (Table 1). The most common primary reason for infertility was unexplained infertility in the follitropin delta group (37/85; 43.5%) and moderate/severe male factor in the follitropin alfa group (21/47; 44.7%). Previous infertility treatment and pregnancies from assisted reproductive technologies were similar between the treatment groups, as were baseline AFC, and serum AMH, estradiol and progesterone levels.

**Table 1:** Demographics and baseline characteristics (full analysis set).

	Follitropin delta starting dose 15 µg/day, *n* = 85	Follitropin alfa starting dose 225 IU/day, *n* = 47	Overall, *n* = 132
Age, years	34.5 ± 4.2	34.5 ± 3.8	34.5 ± 4.1
*Age category*			
<35 years	33 (38.8)	21 (44.7)	54 (40.9)
35–37 years	33 (38.8	15 (31.9)	48 (36.4)
38–40 years	19 (22.4)	11 (23.4)	30 (22.7)
*Race*			
White	84 (98.8)	46 (97.9)	130 (98.5)
Non-white	1 (0.5)	1 (2.1)	2 (1.5)
Bodyweight, kg	66.6 ± 14.0	67.6 ± 12.3	66.9 ± 13.4
BMI, kg/m^2^	24.7 ± 4.88	25.1 ± 4.28	24.8 ± 4.67
*Primary reason for infertility*			
Unexplained infertility	37 (43.5)	11 (23.4)	48 (36.4)
Male factor—Moderate/Severe	22 (25.9)	21 (44.7)	43 (32.6)
Male factor—Mild	15 (17.6)	5 (10.6)	20 (15.2)
Tubal infertility	7 (8.2)	14 (17.0)	15 (11.4)
Endometriosis stage I/II	4 (4.7)	2 (4.3)	6 (4.5)
Primary infertility	65 (76.5)	32 (68.1)	97 (73.5)
Duration of infertility, months	26.4 ± 16.5	27.5 ± 18.3	26.8 (17.1)
Participants with previous IVF/ICSI fertility treatment	17 (20.0)	9 (19.1)	26 (19.7)
Participants with previous clinical pregnancy (ART)	9 (10.6)	7 (14.9)	16 (12.1)
Participants with previous live birth (ART)	7 (8.2)	5 (10.6)	12 (9.1)
Antral follicle count	13.3 ± 5.2	13.9 ± 6.1	13.5 ± 5.5
*Endocrine parameters on stimulation Day 1*			
AMH, pmol/L [ng/mL]	14.1 ± 7.9 [2.0 ± 1.1] [*n* = 85]	16.7 ± 13.3 [2.3 ± 1.9] [*n* = 46]	15.0 ± 10.2 [2.1 ± 1.4] [*n* = 131]
*AMH category*			
<15 pmol/L [<2.1 ng/mL]	52 (61.2)	28 (59.6)	80 (60.6)
≥15 pmol/L [≥2.1 ng/mL]	33 (38.8)	18 (38.3)	51 (38.6)
Missing	0	1 (2.1)	1 (0.8)
Estradiol, pmol/L	141.0 [108.0, 187.0] [*n* = 84]	125.0 [103.0;176.0] [*n* = 47]	136.0 [103.0;187.0] [*n* = 131]
Progesterone, nmol/L	1.0 [0.6; 1.3]	1.0 [0.3; 1.0]	1.0 [0.6; 1.3]

Data are mean ± standard deviation, *n* (%) or median [interquartile range].

Conversion factor for AMH: ng/mL to pmol/L, 7.1429.

AMH, anti-Müllerian hormone; ART, assisted reproductive technology; BMI, body mass index; ICSI, intracytoplasmic sperm injection; IVF, *in vitro* fertilization.

### 3.3 Gonadotropin exposure and concomitant medication

The duration of ovarian stimulation was similar between treatment groups [adjusted mean 9.2 days and 8.9 days for follitropin delta and follitropin alfa, respectively; treatment difference 0.3 (95% CI –0.3; 0.8); Table 2]. Dose increase adjustments were requested for 26 (30.6%) and 10 (21.3%) participants in the follitropin delta and follitropin alfa groups and the mean total dose was 145.5 µg and 150.6 µg, respectively. There were no requests for dose decreases. Slightly more than half of participants received an hCG trigger [49 (57.6%) and 30 (63.8%), respectively].

**Table 2:** Gonadotropin and GnRH antagonist exposure, and trigger for final follicular maturation.

	Follitropin delta starting dose 15 µg/day, *n* = 85	Follitropin alfa starting dose 225 IU/day, *n* = 47
Duration of gonadotropin treatment (days)*	9.2	8.9
Total gonadotropin dose		
µg	145.5 ± 33.1	150.6 ± 24.1
IU	_	2,054 ± 329
Average gonadotropin daily dose, µg/day	15.8 ± 1.2	17.0 ± 1.0
Participants with any gonadotropin dose adjustments		
Increase	26 (30.6)	10 (21.3)
Decrease	0	0
Duration of GnRH antagonist (0.2 µg/day) treatment, days	4.8 ± 1.6	4.5 ± 1.3
Triggering of final follicular maturation		
hCG	49 (57.6)	30 (63.8)
GnRH agonist	36 (42.4)	15 (31.9)
None	0	2 (4.3)

Data are mean ± standard deviation, *n* (%), or adjusted mean.

* Adjusted mean days of stimulation was calculated using an ANOVA model with treatment and AMH group as factors.

GnRH, gonadotropin-releasing hormone; hCG, human choriogonadotropin.

The most common concomitant non-trial medication were sex hormones and modulators of the genital system and were taken by a similar proportion of participants in each treatment group [follitropin delta: 35 (42.4%); follitropin alfa: 20 (42.6%)]—these were predominantly medications to support implantation and had been prescribed according to local practice.

Most participants received an hCG trigger for final follicular maturation in both treatment groups (Table 2).

### 3.4 Efficacy endpoints

The adjusted mean number of oocytes retrieved (primary endpoint) was similar between the follitropin delta and follitropin alfa treatment groups [10.6 and 11.0, respectively; treatment difference –0.3 (95% CI –2.4; 1.7); Table 3]. The distribution of oocytes retrieved is shown in Figure 2, with the category 8–14 oocytes retrieved being the most common category for both treatment groups (Figure 2A), as well as for AMH subgroups (Figures 2C,D). As expected, participants in the AMH <15 pmol/L subgroup had lower numbers of oocytes retrieved than participants in the AMH ≥15 pmol/L. All secondary efficacy endpoints based on ovarian responses were similar between the two treatment groups (Table 3).

**Table 3:** Ovarian responses, endocrine parameters at end of stimulation, fertilization and pregnancy rates (full analysis set).

	Follitropin delta starting dose 15 µg/day, *n* = 85	Follitropin alfa starting dose 225 IU/day, *n* = 47	Treatment difference [95% CI]*
Oocytes retrieved^†^ (primary endpoint)	10.6	11.0	−0.3 [–2.2; 1.7]
Follicles at end of stimulation	14.6 ± 7.4	14.1 ± 6.9	_
Follicles ≥12 mm^†^	12.3	11.8	0.5 [–1.4; 2.3]
Follicles ≥15 mm^†^	9.1	8.9	0.1 [–1.4; 1.6]
Follicles ≥17 mm^†^	6.7	6.3	0.3 [–0.8; 1.5]
Insemination method			
IVF	2 (2.4)	3 (6.4)	_
ICSI	75 (88.2)	41 (87.2)	_
IVF/ICSI	5 (5.9)	0	_
None	3 (3.5)	3 (6.4)	_
MII oocytes	6.1 ± 4.1	6.1 ± 4.3	–
Fertilized MII oocytes relative to oocytes retrieved^†^	5.9	5.7	0.1 [–1.2; 1.5]
Fertilization rate^‡^	55.1%	52.7%	2.4 [–5.7; 10.5]
Blastocysts on Day 5/6^†^	3.7	3.6	0.0 [–0.9; 1.0]
Number of good quality blastocysts on Day 5/6^†^	2.7	2.7	0.0 [–0.8; 0.8]
Participants with blastocyst transfer^#^	67 (78.8)	36 (76.6)	_
Fresh blastocyst transfer	31	15	_
Frozen blastocyst transfer	34	20	_
Missing data	2	1	_
Clinical pregnancy rate per started cycle^§^	34.8%	32.0%	2.8 [–14.3; 20.0]
Implantation rate (blastocyst level)^¶^	46.3%	40.9%	5.5 [–20.1; 31.0]
Endocrine parameters at end of stimulation**			
Estrogen, pmol/L	5,717.2 [*n* = 83]	6,308.6 [*n* = 45]	Ratio 0.9 [0.7; 1.1]
Progesterone, nmol/L	2.7 [*n* = 84]	2.6 [*n* = 45]	Ratio 1.0 [0.8; 1.2]

Data are mean ± standard deviation, or *n* (%), adjusted mean, or median (interquartile range).

* Difference and confidence interval derived using the delta method.

^†^Adjusted mean number was calculated using a negative binomial regression model with treatment and AMH group as factors.

# One participant in the follitropin delta group and one in the follitropin alfa group had two blastocysts transferred, one participant in the follitropin delta group had two nonblastocysts transferred.

‡ The adjusted fertilization rate was calculated using a negative binomial regression model with treatment and AMH group as factors and the number of retrieved oocytes as offset.

¶ Adjusted rate was calculated using a negative binomial regression model with treatment and age group as factors and transferred oocytes as offset.

§ Adjusted clinical pregnancy rate was calculated using a logistic regression model with treatment and age group as factors.

** Adjusted endocrine parameters were calculated using a multiplicative ANCOVA model with treatment and AMH group as factors and baseline serum concentration as covariate.

CI, confidence interval; ICSI, intracytoplasmic sperm injection; IVF, in vitro fertilization; MII, metaphase II.

**Figure 2:**
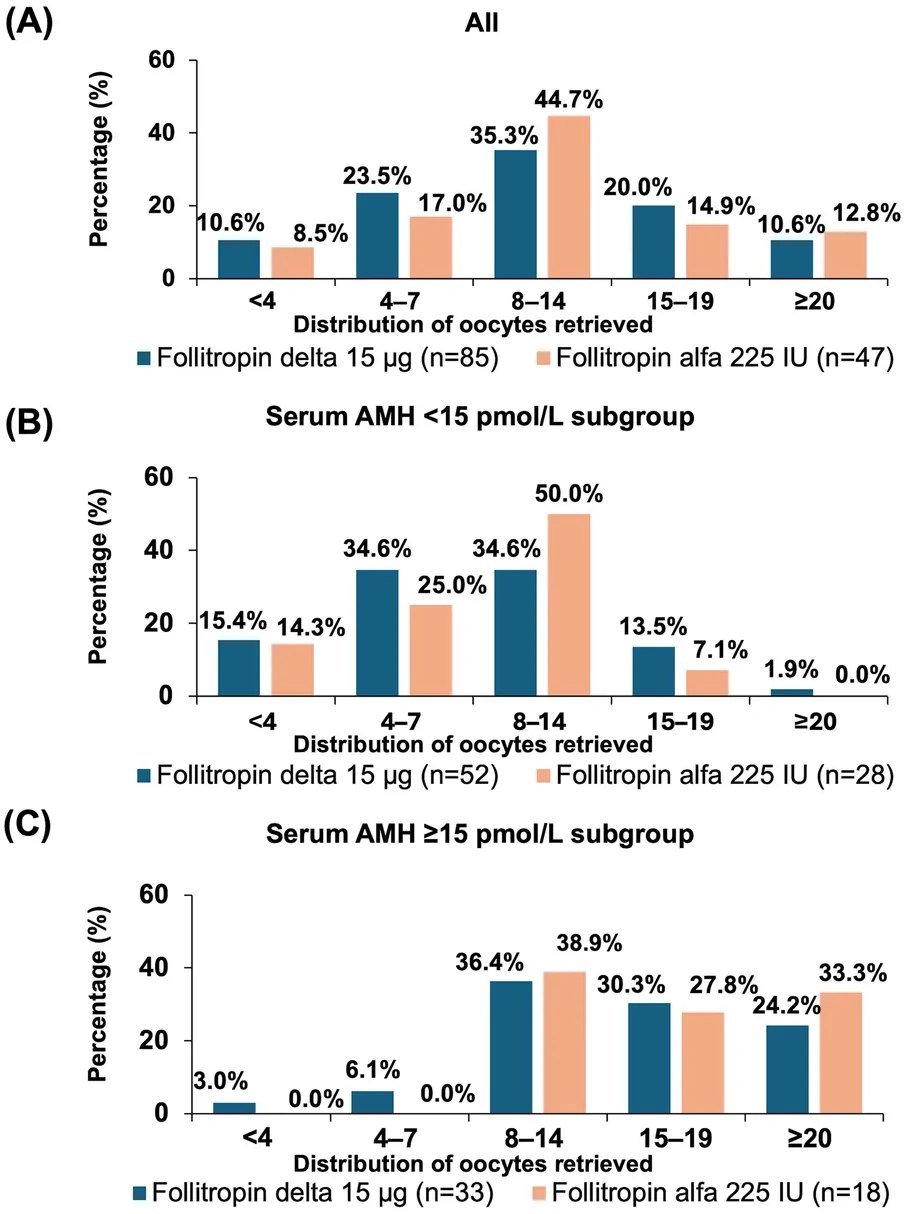
Distribution of oocytes retrieved. **(A)** Overall trial population by treatment group; **(B)** subgroup with serum AMH at Day 1 of stimulation <15 pmol/L; **(C)** subgroup with serum AMH at Day 1 of stimulation ≥15 pmol/L. There was one participant who did not have serum AMH assessed on Day 1 of stimulation. AMH, anti-Müllerian hormone.

### 3.5 Cycle and blastocyst transfer cancellations, and insemination method

There were two participants who did not receive a trigger for final follicular maturation, both in the follitropin alfa group (poor ovarian response, *n* = 1; other reason, *n* = 1). As such, oocyte retrieval was performed in all participants in the follitropin delta group (100%; *n* = 85) and all but two participants in the follitropin alfa group (95.7%; *n* = 45). The choice of IVF, ICSI, or combined IVF/ICSI was determined according to local clinical practice and participant characteristics. Insemination was by ICSI for the majority [116/132 (87.9%)] of participants (Table 3), reflecting clinical practice at the participating Spanish centers and the proportion of participants with male factor infertility.

The first fresh/frozen blastocyst transfer was performed for 78.8% (67/85) and 76.6% (36/47) of participants in the follitropin delta and follitropin alfa groups, respectively. Of the 18 and 11 participants who did not have a fresh/frozen blastocyst transfer within 3 months of the start of stimulation, reasons for no transfer were: no available blastocyst to transfer (follitropin delta, *n* = 10; follitropin alfa, *n* = 3) or “other” reason [follitropin delta, *n* = 8; follitropin alfa, *n* = 8 (including the two participants who did not undergo oocyte retrieval)].

### 3.6 Pregnancy rates

The adjusted clinical pregnancy rates per started cycle were 34.8% for the follitropin delta group and 32.0% for the follitropin alfa group with a treatment difference of 2.8% (95% CI: −14.3; 20.0). The unadjusted clinical pregnancy rates per age category (<35, 35–37 and 38–40 years) declined with increasing age in both treatment arms (Figure 3).

**Figure 3:**
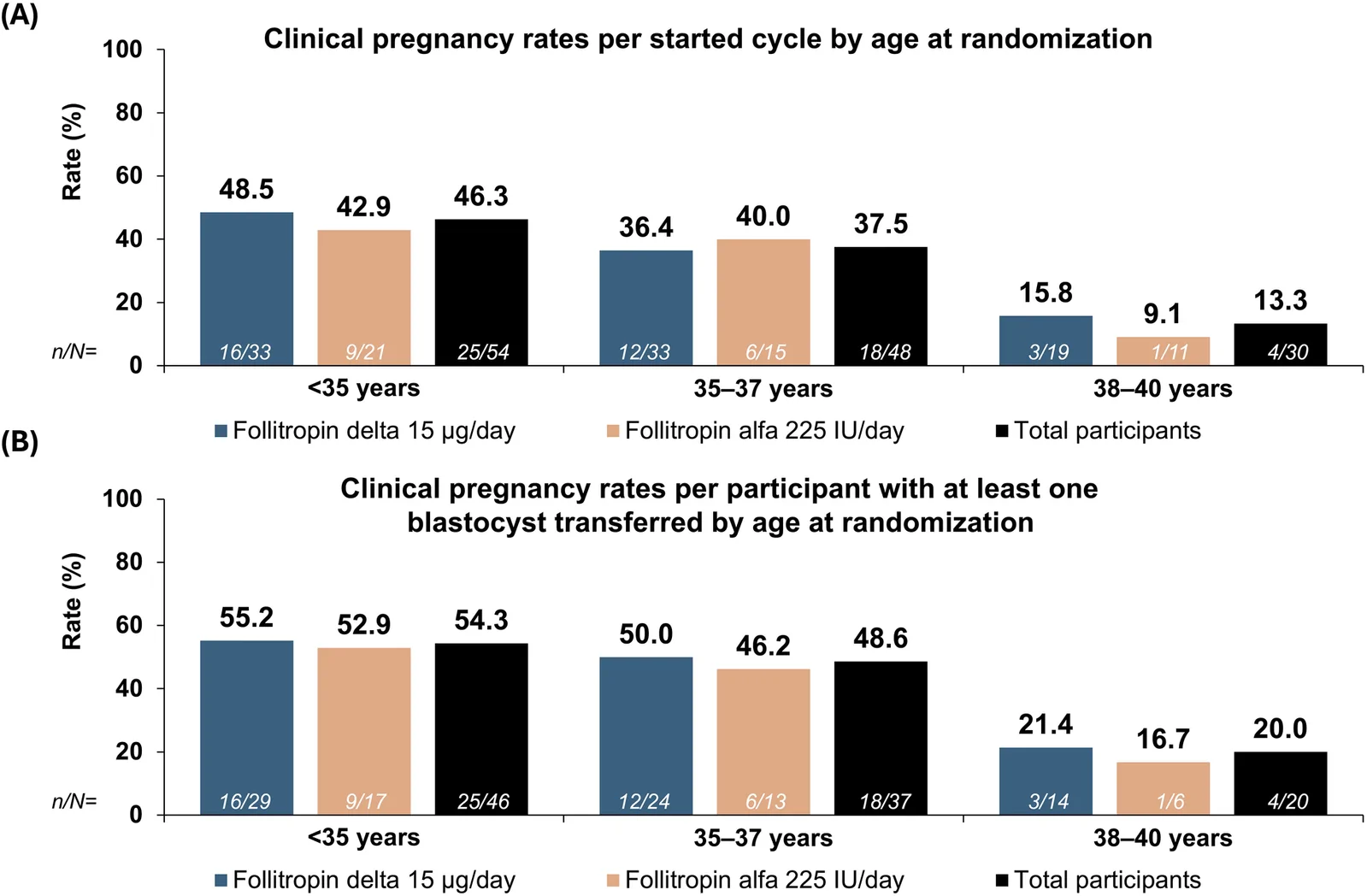
Clinical pregnancy rates from first transfer (either fresh or cryopreserved). All participants with their first blastocyst transfer within 3 months of the start of ovarian stimulation are included. Data presented in the bar charts are unadjusted with *n* = number of participants with a clinical pregnancy and *N* = number of participants who started ovarian stimulation **(A)**; or number of participants with at least one blastocyst transferred **(B)**.

### 3.7 Safety and adverse event monitoring

Adverse events were reported for 28.2% and 42.6% of participants in the follitropin delta and follitropin alfa groups, respectively, with the most common being headache [10.6% (*n* = 9) and 8.5% (*n* = 4)], pelvic discomfort [4.7% (*n* = 4) and 4.3% (*n* = 2)], dysmenorrhea [0% and 6.4% (*n* = 3)], and early OHSS [1.2% (*n* = 1) and 4.3% (*n* = 2); Table 4]. The cases of early OHSS were mild (follitropin delta group, *n* = 1; follitropin alfa group, *n* = 1) or moderate (follitropin alfa group, *n* = 1), graded according to Golan et al. (13), and all three early OHSS cases were in the AMH ≥15 pmol/L subgroup.

**Table 4:** Adverse event and ovarian hyperstimulation syndrome summary (full analysis set).

	Follitropin delta starting dose 15 µg/day, *n* = 85	Follitropin alfa starting dose 225 IU/day, *n* = 47
Participants with any adverse event	24 (28.2)	20 (42.6)
Serious adverse events	0	2 (4.3)
Adverse drug reactions	9 (10.6)	7 (14.9)
Serious adverse drug reactions	0	0
Adverse events leading to discontinuation	0	1 (2.1)
Adverse events leading to death	0	0
*Adverse events occurring in ≥4% of participants in either treatment group*		
Headache	9 (10.6)	4 (8.5)
Pelvic discomfort	4 (4.7)	2 (4.3)
Dysmenorrhoea	0	3 (6.4)
Early ovarian hyperstimulation syndrome	1 (1.2)	2 (4.3)
Grade 1 or 2 (mild)	1 (1.2)	1 (2.1)
Grade 3 (moderate)	0	1 (2.1)
Breast swelling	0	2 (4.3)
Pelvic pain	0	2 (4.3)
Fatigue	0	2 (4.3)
Haemoperitoneum	0	2 (4.3)
*Preventive intervention for potential early ovarian hyperstimulation syndrome*		
Any preventive intervention	17 (20.0)	11 (23.4)
Cycle cancellation	0	0
Triggering with GnRH agonist	17 (20.0)	11 (23.4)
Administration of dopamine agonist	1 (1.2)	0

Data are *n* (%).

GnRH, gonadotropin-releasing hormone.

Preventive interventions for potential early OHSS included triggering of final follicular maturation with a GnRH agonist instead of hCG, the administration of a dopamine agonist, and use of a freeze-all strategy with deferred cryopreserved transfer when clinically indicated. Preventive interventions were administered to 20.0% [17/85] and 23.4% [11/47] of participants in the follitropin delta and follitropin alfa groups, respectively (Table 3), the majority of whom were in the AMH ≥15 pmol/L subgroup (*n* = 14 and *n* = 9, respectively). All five participants in the AMH <15 pmol/L subgroup who received preventive intervention were given a GnRH agonist trigger as the preventive intervention. There were no transfer cancellations as a preventive treatment for potential early OHSS as deferred cryopreserved transfers were permitted for participants perceived to be at risk of OHSS. There were no cases of late OHSS among either treatment group.

## 4 Discussion

Here, we present an exploratory *post hoc* analysis of trial results for women seeking medical support to become pregnant at specialist reproductive medicine centers in Spain who underwent ovarian stimulation with either conventionally dosed follitropin delta 15 µg/day or follitropin alfa 225 IU/day for the first 5 days, after which dose adjustments could be made. Participants enrolled in Spain are of particularly interest because Spain is the largest provider of ART services in the Europe, and trial drug doses represent the upper limit of the commonly used rFSH dose for women who are predicted to have a normal ovarian response (3). These data are important to understand the ovarian responses and pregnancy rates from the first transfer (fresh or frozen) with the higher starting dose of follitropin delta 15 µg/day and follitropin alfa 225 IU/day among infertile women and women with a male partner diagnosed with infertility. These findings should be interpreted in the context of the trial eligibility criteria as all participants were judged by the investigator to be suitable for a gonadotropin starting dose of 225 IU meaning that predicted hyperresponders were not suitable for enrolment. Specifically, we excluded patients who were judged to have been at risk of OHSS, had a prior exuberant ovarian response to gonadotropins, or had PMOS (previously known as PCOS). As such, the results are not generalizable to all IVF/ICSI patients.

In our study, an average adjusted yield of approximately 11 oocytes was obtained per participant in both treatment arms, and an adjusted clinical pregnancy rate was 34.8% and 32.0% per started cycle for follitropin delta and follitropin alfa, respectively, for the first transfer within 3 months of starting ovarian stimulation. Understanding the pregnancy rate from the first embryo transfer per cycle provides a realistic baseline for success, highlighting the potential of specific protocols being used in the IVF/ICSI cycle. Comparable efficacy outcomes were observed between follitropin delta 15 µg/day and follitropin alfa 225 IU/day, and similarly, rates of OHSS were also similar between the two treatment groups. Preventive interventions for early OHSS were mostly the use of a GnRH agonist trigger along with a freeze-all strategy; however, as only 36 patients in the follitropin delta group and 15 patients in the follitropin alfa group received a GnRH agonist trigger, further data are required to elucidate any treatment differences in the number of oocytes retrieved, pregnancy rates and OHSS risk for follitropin delta and follitropin alfa when using either an hCG or GnRH agonist trigger for final follicular maturation in a GnRH antagonist protocol.

In Spain, fertility specialists understand that reproductive success (i.e., a live birth) is not negatively impacted by opting for a cryopreserved transfer instead of a fresh transfer (14). Clinicians in Spain often consider frozen embryo transfer (FET) the safest strategy for patients with a high ovarian response, as OHSS is not associated with FET cycles and this approach does not compromise clinical pregnancy or live birth rates (15). Patients with regular menstrual cycles like those in our study, are usually prepared for frozen embryo transfers using modified natural cycles (hCG and luteal support), unless contraindicated, oppose to using substituted cycles (16). A flexible approach to scheduling a frozen-thawed blastocyst transfer is possible using a modified natural cycle, and is likely to reduce costs and the treatment burden without compromising the chance of a live birth (17).

In clinical practice, a threshold of 18–19 follicles ≥11 mm in diameter is normally used to decide whether to use a GnRH agonist; however, in our trial, a higher threshold was used of 25 or more follicles with ≥12 mm diameter. As such, it is possible that clinicians may choose to apply preventive measures for early OHSS in a higher proportion of patients who are given a starting dose of follitropin delta 15 µg or follitropin alfa 225 IU than we observed in our study, particularly if they are more cautious and use lower thresholds of follicles to guide their decision-making (18).

In our study, there were no dose decrease adjustments for follitropin delta or follitropin alfa—all dose adjustments were dose increases, which reflects the well-selected population who were suitable for the starting dose of follitropin delta 15 µg/day or follitropin alfa 225 IU/day. In the ADAPT-1 trial, the vast majority of requests for dose adjustments were also for a dose increase (5). Given the lack of evidence on the value of dose adjustments during ovarian stimulation, it is important that the gonadotropin starting dose is appropriate based on patient characteristics and desired outcome (3). As such, taking into account the ovarian responses, low rate of early OHSS and the good pregnancy rates, the participants in our study appear to have been appropriately selected for a starting dose of follitropin delta 15 µg or follitropin alfa 225 IU.

Before the start of the trial, previous analysis of >1,500 clinical trial participants suggests that follitropin delta 10 µg/day and follitropin alfa 150 IU/day provide similar ovarian responses (19). However, at the time of the trial the approved daily dose of follitropin delta was determined according to its dosing algorithm based on serum AMH and bodyweight, with a maximum dose of 12 µg/day for the first cycle of ovarian stimulation and 24 µg/day for subsequent cycles. In our current trial, about 80% of the trial population were naïve to ovarian stimulation, meaning that if they had received the approved follitropin delta dose according to the dosing algorithm, the maximum dose these patients could have received was 12 µg/day, not the 15 µg/day dose received in this trial. In Spain, a gonadotropin 225 IU starting dose is commonly used for women with a predicted normal ovarian response for an ovarian stimulation cycle; the 225 IU starting dose is often chosen over 150 IU in order to maximize oocytes yield and embryos which then can be cryopreserved, which would then bring down the overall cost per frozen oocyte or embryo, even if the pregnancy and live birth rate from the first transfer are similar (20, 21). Furthermore, >15 oocytes retrieved is associated with an increase in cumulative pregnancy rates but plateaus at 21–25 oocytes retrieved (20–22).

### 4.1 Challenges, limitations and future directions

A key limitation is that this study represents a *post hoc* subgroup analysis of a larger trial and only included participants enrolled in Spain. The study was not independently powered for formal efficacy comparisons or detailed subgroup analyses. Our findings should therefore be interpreted as exploratory and hypothesis-generating.

Although female age is an important determinant of ART outcomes, this Spanish subgroup analysis was not independently powered for detailed age-stratified comparisons. Nonetheless, we included age group as a factor in the logistic regression model for clinical pregnancy rates, and descriptive age-category outcomes are presented; however, additional age-stratified analyses have not been conducted because of the limited subgroup sample size. Similarly, we did not assess reproductive outcomes separately for Day 5 vs. Day 6 blastocysts as the analysis was not powered for this comparison.

Among the trial participants, there was a higher proportion with severe male factor infertility in the follitropin alfa group (44.7%) compared with the follitropin delta group (25.9%). Importantly, the impact on fertilization and downstream embryology outcomes is substantially mitigated by the predominant use of ICSI among 87.9% of participants overall, which is the established fertilization method for male factor infertility, as well as being the preferred fertilization method if preimplantation genetic testing is planned (23). As the fertilization rates (55.1% vs. 52.7%), mean number of blastocysts on Day 5/6 (3.7 vs. 3.6), and mean number of good-quality blastocysts on Day 5/6 (2.7 vs. 2.7) were comparable between the treatment arms, as were clinical pregnancy rates (34.8% vs. 32.0% per started cycle), it is unlikely that the imbalance between treatment groups of severe male factor infertility as the primary reason for infertility adversely biased efficacy outcomes.

In addition, further data are required to determine whether specific local clinical practices in Spain affect the number of oocytes retrieved and clinical pregnancy rates. We also did not monitor drug wastage (i.e., unused drugs left over in the pre-filled injection pens); however, both follitropin delta and follitropin alfa are available in pre-filled pens, with different quantities of the drug to minimize wastage.

## 5 Conclusions

Our exploratory findings show that higher starting doses of follitropin delta 15 µg/day can be successfully used in appropriately selected infertile patients in Spain who are considered suitable for a conventional 225 IU/day gonadotropin starting dose, resulting in a good average oocyte yield and first-transfer clinical pregnancy rates, while maintaining a low incidence of early OHSS. These data will support clinicians to understand the follitropin delta microgram unit compared to gonadotropins dosed in IU.

## Data Availability

The datasets presented in this article are not readily available because the data are owned by the trial sponsor. Requests to access the datasets should be directed to rita.lobo@ferring.com.
