# Supplementary material for: Comparative outcomes of conventional follitropin delta 15 µg/day versus follitropin alfa 225 IU/day for ovarian stimulation: results from an exploratory post hoc analysis of Spanish participants from the ADAPT-1 randomized controlled trial
Source: Front Reprod Health. 2026 Sep 30;8:1927550. doi: 10.3389/frph.2026.1927550 (PMC13662129; doi:10.3389/frph.2026.1927550)
Supplement: Supplementary file 1 [file Table1.docx]

## **Table S1. ADAPT-1 trial inclusion and exclusion criteria (Spanish subgroup analysis)**

| **Inclusion criteria** | **Exclusion criteria** |
| --- | --- |
| Patients had to meet all of the criteria listed below to be eligible for participation in the trial.   1. Informed Consent Form signed prior to screening evaluations. 2. In good physical and mental health. 3. Pre-menopausal females between the ages of 18 and 40 years. The patients must be at least 18 years (including the 18th birthday) when they sign the informed consent and no more than 40 years (up to the day before the 41st birthday) at the time of randomization. 4. Infertile women diagnosed with tubal infertility, unexplained infertility, endometriosis stage I/II or with partners diagnosed with male factor infertility, eligible for in vitro fertilization (IVF) and/or intracytoplasmic sperm injection (ICSI) using fresh or frozen ejaculated sperm from male partner or sperm donor. 5. Infertility for at least one year before randomization for patients ≤37 years or for at least 6 months for patients ≥38 years (not applicable in case of tubal or severe male factor infertility). 6. Regular menstrual cycles of 21-35 days (both inclusive), presumed to be ovulatory. 7. Transvaginal ultrasound documenting presence and adequate visualization of both ovaries, without evidence of significant abnormality (e.g. no endometrioma greater than 3 cm, and no enlarged ovaries or ovarian cyst not due to polycystic ovarian syndrome, which would contraindicate the use of gonadotropins) and normal adnexa (e.g. no hydrosalpinx) within 1 year prior to randomization. Both ovaries must be accessible for oocyte retrieval. Early follicular phase (cycle day 2-4) serum levels of FSH between 1 and 15 IU/L (results obtained within 3 months prior to randomization). 8. Enrolment at a Spanish trial site. | Patients who met any of the criteria listed below were not eligible for participation in the trial.   1. Primary ovarian failure. 2. Known endometriosis stage III-IV. 3. Considered unsuitable for controlled ovarian stimulation with a dosing regimen corresponding to approximately 225 IU/day gonadotropin, as judged by the investigator. 4. History of previous episode of OHSS or exuberant ovarian response to gonadotropins, and polycystic ovarian syndrome. 5. One or more follicles ≥10 mm (including cysts) observed on the transvaginal ultrasound prior to randomization on stimulation day 1 (puncture of cysts is allowed prior to randomization). 6. Any known endocrine or metabolic abnormalities (pituitary, adrenal, pancreas, liver or kidney) which can compromise participation in the trial with the exception of controlled thyroid function disease. 7. Known tumours of the ovary, breast, uterus, adrenal gland, pituitary or hypothalamus which would contraindicate the use of gonadotropins. 8. Fibroid tumours of the uterus incompatible with pregnancy. 9. Currently breast-feeding. 10. Undiagnosed vaginal bleeding. 11. Findings at the gynaecological examination at screening which preclude gonadotropin stimulation or are associated with a reduced chance of pregnancy, e.g. congenital uterine abnormalities or retained intrauterine device. 12. Pregnancy (negative urinary pregnancy tests must be documented at screening and prior to randomization) or contraindication to pregnancy. 13. Use of fertility modifiers during the last menstrual cycle before randomization, including dehydroepiandrosterone (DHEA) or cycle programming with oral contraceptives, progestogen or oestrogen preparations. 14. Hypersensitivity to any active ingredient or excipients in the medicinal products used in the trial. 15. Previous participation in the trial. 16. Use of any non-registered investigational drugs during the last 3 months prior to randomization. |
